# Mechanochromic cholesteric liquid crystal devices for mechanical strain detection

**DOI:** 10.1038/s41598-026-37723-4

**Published:** 2026-01-27

**Authors:** Francisco Sousa, João Santos, José F. Malta, João P. Canejo, Ana P. C. Almeida, Pedro L. Almeida

**Affiliations:** 1 i3N/CENIMAT, Department of Materials Science, NOVA School of Science and Technology, NOVA University Lisbon, Campus de Caparica, Caparica, 2829 - 516 Portugal; 2 LAQV REQUIMTE, Chemistry Department, NOVA School of Science and Technology | NOVA FCT, Portugal, Caparica, 2829-516 Portugal; 3 Mechanical Engineering Department, UnIRE/ISEL, ISEL, Polytechnic University of Lisbon, Rua Conselheiro Emídio Navarro, 1, Lisbon, 1959-007 Portugal; 4 Portalegre Polytechnic University, IPPortalegre, Praça do Município, 11, 7300-110 Portalegre, Portalegre, Portugal

**Keywords:** Cholesteric liquid crystal, Elastomer, Polymer matrix, Structural colour, Mechanochromic, Smart sensor, Chemistry, Engineering, Materials science

## Abstract

The properties of functional soft materials have opened the doors for the development of a wide range of stimuli-responsive smart devices. The ability to react to factors such as humidity, temperature or pressure, inducing changes in the materials’ properties, for instance, colour, constitutes the basis of smart sensing. For such applications, liquid crystals (LCs) present a particularly compelling and innovative class of materials that have been extensively studied. Amongst LCs, cholesteric LCs (CLCs) have been gaining much attention for different applications, owing to their selective and tuneable reflection of circularly polarised light. This work was focused on the application of CLC-based smart sensing devices for mechanical strain detection through the production of mechanical stress-sensitive devices. These sensors may be used, for instance, as standalone sensors to evaluate a crack propagation in concrete structures, having a visual readout. For this effect, CLC elastomer (CLCE)-based mechanochromic devices were produced. PDMS was embedded with 3D CLCE structures, obtained through the crosslinking of an acrylate-terminated CLCE (ACLCE) precursor solution, followed by shaping either by dropwise addition onto a silicon oil bath – with and without agitation – or by moulding. The sensorial capabilities of the devices were tested by subjecting them to mechanical deformation to assess whether this leads to a change in colour. It was concluded that, considering the main goal of achieving mechanochromic behaviour, the ACLCE structures obtained from dropwise addition, under no agitation, exhibited the strongest and most uniform mechanochromism.

**Supplementary Information:**

The online version contains supplementary material available at 10.1038/s41598-026-37723-4.

## Introduction

Despite the high impact and contribution of functionally static hard materials for economic development, there is an ever-growing interest in the development of different classes of materials. Soft materials constitute one such class of materials, relevant for their dynamic and versatile functionality, despite their reduced strength and durability when compared to hard materials^1^.

Soft materials have opened the doors to the development of advanced smart devices, as is the case with soft actuators, which are flexible devices with the capability to move and/or control a machine or a system, overcoming the drawback of low work and power density associated with standard actuators^2^ with the potential to be employed in a wide variety of applications, such as robotics^3^. Furthermore, soft actuators can also be applied to sensorial applications. Such is the case of the work developed by Zhao et al.^4^ on which the authors developed a porous poly(ionic liquid) (PIL) membrane actuator with the capability of bending in the presence of low amounts of organic solvents with high sensitivity.

In this sense, different soft materials have been studied and developed, such as polymers, gels, and biomaterials. However, when it comes to stimuli-responsive smart soft materials, liquid crystals (LCs) are especially promising^1^. LCs can be classified as materials that exhibit a liquid state with optical and dielectric anisotropies and belong to a state of matter between an isotropic liquid and a crystalline solid, combining the fluidity of a liquid with the anisotropy of a solid^1,5^. Furthermore, LCs present a set of characteristics that make them highly appealing materials, such as birefringence, high transparency in the visible range, and the capacity to be modulated^6^.

**Fig. 1:**
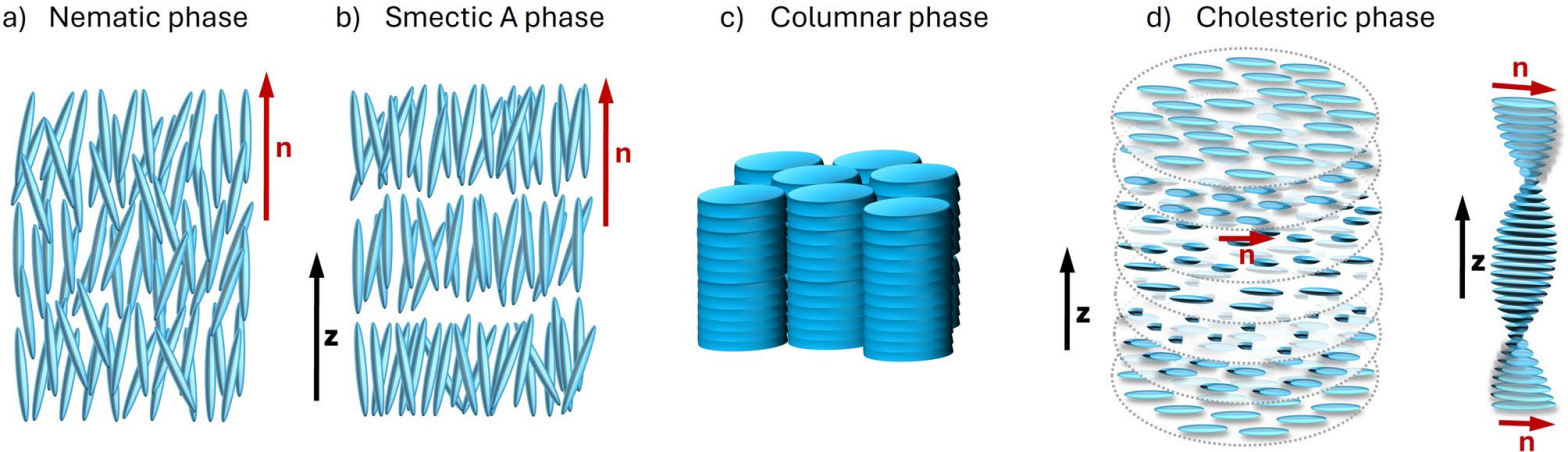
Illustration of (**a**) a nematic LC, (**b**) a smectic A LC, (**c**) a columnar LC, and (**d**) a cholesteric LC.

LCs can be classified in different manners, such as, according to the method by which the orientational order of the crystalline solid is broken, LCs can be classified as thermotropic if the mesophase is formed through temperature variation, and lyotropic if obtained through concentration variation. Also, LCs can be classified according to their molecular arrangement, as nematic (Fig. 1a) when they possess long-range orientational order, but no positional order, smectic (Fig. 1b) when they possess both orientational and translational order, columnar (Fig. 1c) when the molecules align themselves in cylindrical structures, and cholesteric (Fig. 1d) formed by chiral molecules or when chiral compounds are incorporated into the nematic [7].

**Fig. 2:**
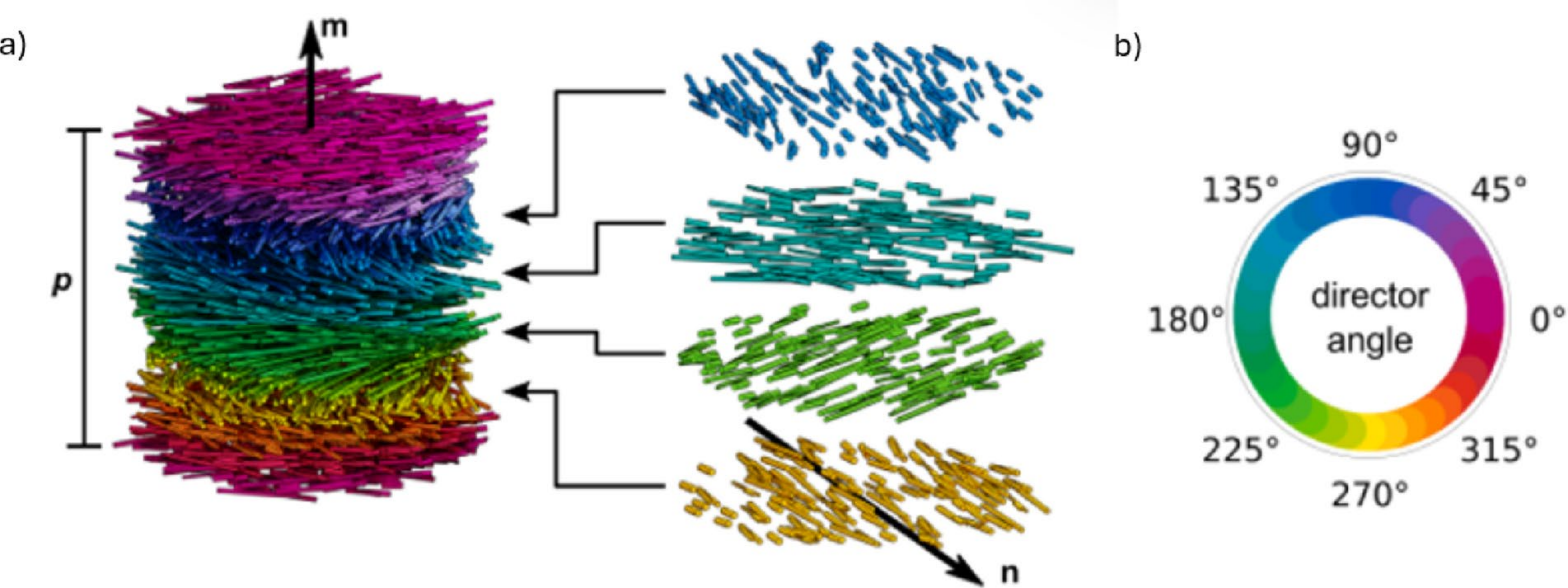
(**a**) Schematic of the structure of CLCs; (**b**) Respective director angle colour coding (n – director, m – helical axis, p – pitch). (Adapted from^8^).

Among the LC phases, cholesteric liquid crystals (CLCs) are of special interest due to their selectivity to wavelength and polarisation^7^. The cholesteric phase is characterised by a self-organisation into a helicoidal configuration, where the director (*n*), which describes the average molecular orientation, is perpendicular to the helical axis (*m*). CLCs can be characterised by their pitch (*p*), which corresponds to the distance over which the director rotates 360 ° (Fig. 2)^7^.

Indeed, it is the helicoidal configuration of the CLCs that confers them with their interesting properties – when in a planar texture state, CLCs can reflect polarised light with the same handedness of the helical axis and transmit light with different handedness^9^. Furthermore, considering that only wavelengths between the wavelength range (Δλ), which is dependent on the anisotropy of the refractive index Δ*n* (Δλ = *p*Δ*n*) are reflected (while wavelengths outside this range are transmitted), and that the wavelength of reflected light is also dependent on the angle of incidence (*θ*) according to the Bragg’s equation – *λ(θ) = npcos(θ)* – and considering that the pitch is dependent on multiple factors, such as temperature, electric field, mechanical deformation and nature and concentration of impurities, it is possible to modulate the CLCs, adjusting the reflected wavelength, and therefore adjusting the exhibited interference colour^5,10^.

When it comes to CLCs, two major drawbacks arise – narrow reflection bandwidth and fluidic liquid crystal instability^11^. In this sense, stabilisation of the CLCs can come as a way to overcome such drawbacks, which can be achieved by: (1) dispersing the CLCs in a polymer network, forming polymer-dispersed CLCs (PDCLCs), which retain the properties of the CLCs, while conferring higher stability, coherence, utility, and flexibility, especially in structures with inhomogeneous pitch distributions^11,12^, or (2) working with polymeric CLCs in the glassy or crosslinked state, such as CLC elastomers (CLCEs), which combine the orientational order of LCs with the rubbery elasticity of polymer networks, allowing the reduction of the pitch through CLCE thinning, caused by mechanical deformation, leading to structural colour changes^5,13,14^.

In fact, CLCs, due to their self-assembled helix superstructures, as well as the ease of fabrication of 1D photonic crystal (PD) structures, present a particularly interesting type of stimuli-responsive material for sensorial applications^15^. Geng et al.^10^ developed CLCE fibres, based on an acrylate-terminated liquid crystalline oligomer, prepared through a thiol–acrylate Michael addition, with excellent mechanochromic behaviour encompassing the whole visible range, upon being submitted to mechanical stress, with elongation strain up to 200%. Baleko et al.^16^ developed a plasticised polyvinyl alcohol (PVA)-based PDCLC device with photo/mechanochromic behaviour, which exhibited a blue shift when exposed to UV light, due to the reduction of the twisting power of the chiral-photochromic dopant, and a change in optical properties, when mechanically stretched.

Monitoring cracks and crack growth rates is an important part of structural health monitoring for concrete infrastructure, and several human and automatic monitoring approaches have arisen over time. It was already proposed by Camo et al. the usage of cholesteric liquid crystal elastomers (CLCEs) applied as a coating for the detection of new cracks and monitoring of their progression (a colour change is perceptible since the reflected wavelength if proportional to the cholesteric pitch and that the pitch is strain sensitive). A crack formation or progression can be easily detected thanks to a change in colour^17^. Indeed, the development of sensors can present a high social and economic impact, by overcoming different issues, such as the visual identification of strain due to mechanical stresses, which might inform about the existence of excessive strain. The sensors herein presented may be used as standalone strain sensors, which may be used to evaluate concrete crack’s width evolution or its growth rate, without requiring any other accessories, equipment, or energy source, as required by other available options^18^. The present work focused on the development of strain detection CLC-based devices for smart sensing of mechanical stress. For this effect, mechanochromic CLCE-based 3D structures, obtained through crosslinking of an acrylate-terminated CLCE precursor solution, followed by either shaping through dropwise addition in an immiscible medium (silicon oil), or by shaping with PLA moulds, were obtained. The obtained sensors can be glued to a concrete surface and serve as a way to evaluate crack propagation. The usage of spheres dispersed in PDMS allows the reduction of the amount of CLC present in each sensor, thus reducing the individual cost of the sensors. These sensors have a visual readout, the change of reflected colour, and do not require a power supply or other equipment to be used.

## Materials and methods

### Synthesis of acrylate-terminated CLC elastomers

The synthesis of the acrylate-terminated CLC elastomers (ACLCEs) was based on a thiol–acrylate Michael addition reaction and was conducted in a vial sealed with a Suba-Seal, submerged in a silicone oil bath (350 cSt; *Xiameter*), kept at 80 °C, and the successive steps were performed under stirring at approximately 250 rpm. Adapting the procedure described by Geng et al.^10^ diacrylate monomer 1,4-bis-(4-(3-acryloyloxypropyloxy)benzoyloxy)−2-methylbenzene (RM257; *BLD Pharm;* 𝑀_𝑛_ = 588.60) was dissolved in toluene to achieve a concentration of 0.8073 g/mL. (3R,3aS,6aS)-hexahydrofuro[3,2-b]furan-3,6-diylbis(4-(4-((4(acryloyloxy)butoxy)carbonyloxy)benzoyloxy)benzoate) (LC756; *Synthon Chemicals;* 𝑀_𝑛_ = 966.89), which acts both as a monomer and as the chiral dopant, and the photo-initiator 2,2-Dimethoxy-2-phenylacetophenone (Irgacure 651; *Sigma-Aldrich;* 𝑀_𝑛_ = 256.30) were added to reach a concentration of 0.0536 g/mL and 0.0202 g/mL, respectively. The solution was then stirred for 1 h. Following this, dithiol chain extender 2,2’-(Ethylenedioxy)diethanethiol (EDDET; *BLD Pharm;* 𝑀_𝑛_ = 182.30*)* was added and stirred for 10 min to achieve a concentration of 0.18 mL/mL relative to the amount of toluene. In the end, a solution of triethylamine (TEA; *CarloErba;* 𝑀_𝑛_ = 101.19) and toluene of [1:10] (V/V), which acts as a crosslinking catalyst, was added dropwise in 6 s intervals for 15 min, to achieve a concentration of 0.17 mL/mL relative to the amount of toluene at the time of addition. The vials were then wrapped in aluminium foil to guarantee heat retention.

### Shaping of the acrylate-terminated CLC elastomers

The ACLCE shaping process was based on the production of emulsions through the agitation of two immiscible liquids and on the anisotropic deswelling technique for cholesteric self-assembly. For this effect, the ACLCE precursor solution was first transferred to a syringe with a dispensing tip, followed by the dropwise addition into an oil bath, to leverage not only the interfacial tension for shape formation, but also the solvent evaporation for the cholesteric phase formation and for hardening of the formed microspheres^19,20^.

Different conditions were applied in this step: (a) magnetic stirring at 100 rpm; (b) mechanical stirring (*Heidolph Hei-TORQUE Expert 200* overhead stirrer) with a polytetrafluoroethylene (PTFE) pitched-blade impeller to generate an axial flow, at 40 rpm (positioned at 5 mm above the bottom of the container); (c) mechanical stirring with a custom 3D-printed polylactic acid (PLA) anchor-type impeller to generate a tangential flow, at increasing rotations, starting at 10 rpm with step of 6 rpm per droplet that reached the bottom of the container, until reaching 70 rpm (positioned at 2 mm above the bottom of the container); (d) free-fall dropwise addition with no stirring. The obtained structures were kept in silicon oil for 1 day and were then dried with paper towels.

When producing the acrylate-terminated CLC elastomer by the dropwise addition method, as stated in the Supporting Information, the magnetic stirring method was discarded due to the coalescence of the spheres. The employed mechanical stirring also presented some drawbacks (described in the Supporting Information), which were overcome.

After the production of the beads, the ss-ACLCE beads were washed with water and soap to remove the silicon oil and were afterwards reswelled with toluene and dried (as described in the Supporting information), to recover their mechanochromic behaviour that was hindered by the previous washing.

After these steps, single ss-ACLCE beads were embedded in a double-layer PDMS structure to assess the mechanochromic response of the CLCs.

### Embedding of PDMS with ACLCEs

Single ACLCE beads obtained from free-fall dropwise addition were placed on top of PDSM films with different ratios of silicone elastomer base to curing agent – [10:0.5], [10:0.67], [10:0.84], and [10:1.0]. PDMS was tinted with black China ink (Vallejo) at a concentration of 7% w/w, and cured at 50 °C for 1 day, to increase the contrast for the mechanochromism tests. The single bead was then embedded in a final PDSM layer without tinting, which was then cured at 50 °C for 1 day.

### Characterization

The mechanochromic behaviour was analysed by measuring the reflected with an *Olympus BX51* optical microscope coupled with a monochromator and equipped with an *Olympus KL2500* LCD light source, while submitting the samples to incrementally increasing mechanical stimulation either through the use of calibrated weights, or through the use of a *MINIMAT* - Mini-Mechanical Testing System (firmware version 3.1) equipped with a 20 N load cell, configured for uniaxial tensile testing, with an elongation rate of 0.5 mm/min.

Using the same method as Ying et al. to ensure constant humidity conditions^21^, the tests were carried out inside a dry chamber, where humidity was controlled using silica gels and was kept at approximately 40% hmidity.

## Results and discussion

### ACLCE semi-spheres

**Fig. 3:**
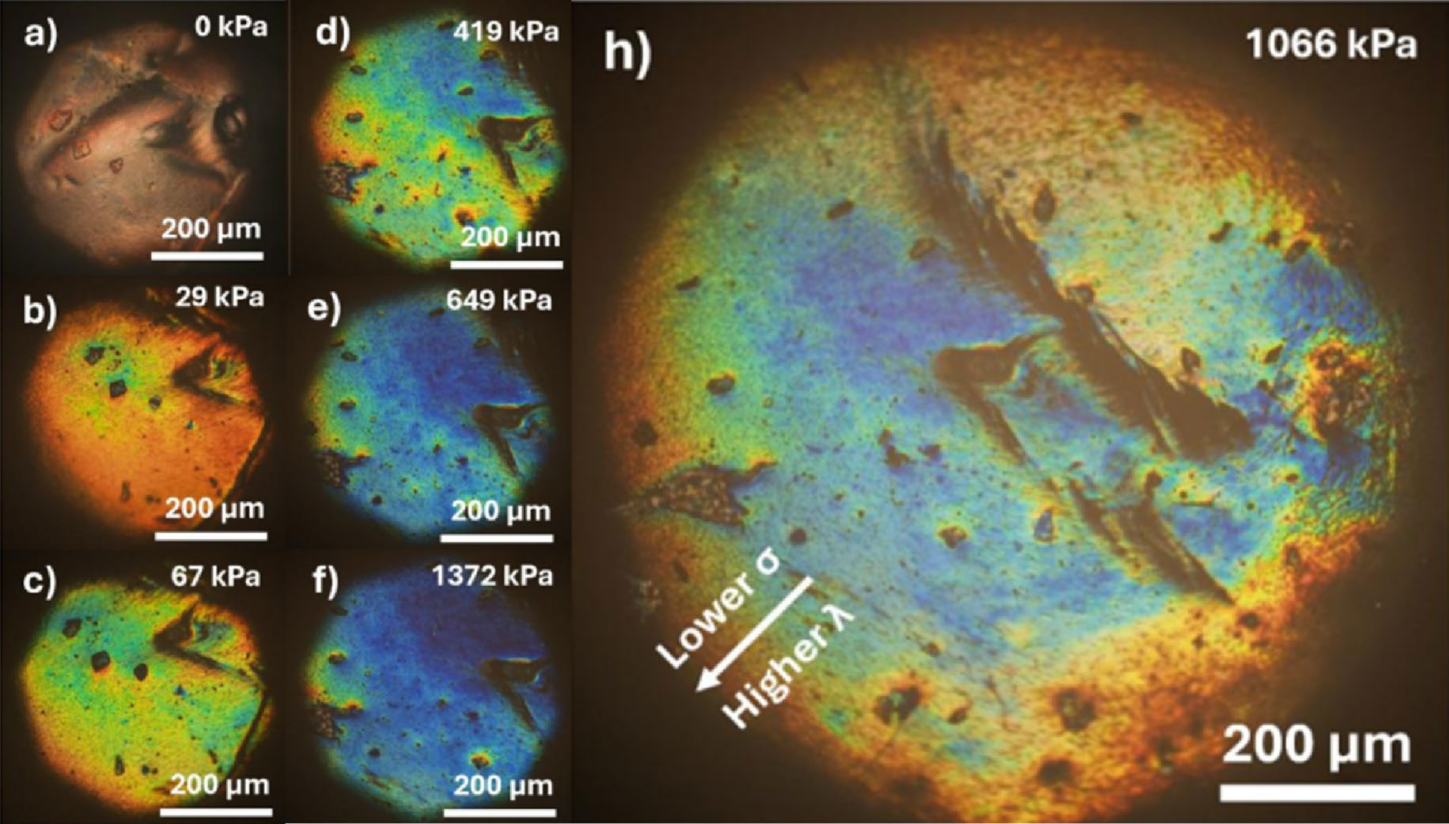
Optical microscopy images in reflection mode with parallel polarisers, demonstrating the mechanochromic response of ss-ACLCEs, when submitted to incremental pressure values; At 1066 kPa, the colour gradient generated by an uneven stress throughout the surface of the ss-ACLCE is easily visualised.

Although all the utilised production methods yielded ACLCE-based structures of similar size, the free-fall dropwise addition method led to the narrowest size distribution. Furthermore, out of the ACLCE-based structures, only the ss-ACLCEs obtained through the free-fall dropwise addition method had their mechanochromic behaviour measured. When analysed through optical microscopy, it was possible to observe that, when no stimulus was applied, the ss-ACLCEs exhibited red selective reflection (Fig. 3a). However, when incremental values of pressure were applied to the sample, a shift to lower wavelengths was observed, due to the helix pitch reduction (Fig. 3a and h).

Furthermore, it was also possible to observe that the distribution of the reflected wavelength was not uniform throughout the area of the ss-ACLCE sample. This effect was particularly visible in Fig. 3h, where the edges presented red reflection, which gradually shifted to blue the closer it got to the centre of the bead. This effect occurred due to the spherical shape of the ACLCE sample. When compression was first applied, the only point of contact occurred in the centre of the semi-sphere, and, with the increase of pressure, the contact area increased, leading to higher deformation in the centre when compared to the edges of the contact area, leading to the observed gradient.

**Fig. 4:**
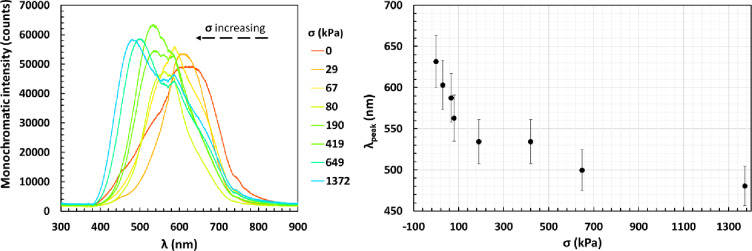
(**a**) Spectra demonstrating the mechanochromic response of ss-ACLCEs, when submitted to incremental pressure values; (**b**) Peak reflected wavelength (λ_peak_) as a function of applied stress, σ.

For each of the applied pressures, the spectra depicting the reflected light, in the visible range, were obtained (Fig. 4a). As it can be seen, it was possible to achieve a shift in wavelength from around 630 to 480 nm. Overall, the ss-ACLCEs showed a pronounced mechanochromic response, demonstrating the high potential of these devices as sensors for applications such as concrete crack propagation evaluation.

Furthermore, by plotting the values of the peak wavelength with the applied pressures (Fig. 4b) it was observed that the reflected wavelength presented a nonlinear dependence on the applied pressure, which occurred due to the gradual increase in contact area. The required pressure necessary to further deform the sample increases with the increase in contact area, hence the non-linear relationship.

### PDMS-based ss-ACLCE structure

For a fully functional strain sensor, the active sensorial component — the CLCs — must be embedded in a protective matrix, enabling its practical application in civil structures. For this study, single ss-ACLCE beads were embedded on a double-layer PDMS structure.

Indeed, PDMS/ACLCE composites with different silicon elastomer base to curing agent ratios were tested – 10:0.5, 10:0.67, 10:0.84, and 10:1.0 – and the respective spectra, under tensile strain, were obtained. However, due to the intensification of the background light source caused by the optical transmittance of PDMS, the main spectral peaks obtained in optical microscopy were overlapped with the background, at around 590 nm.

**Fig. 5:**
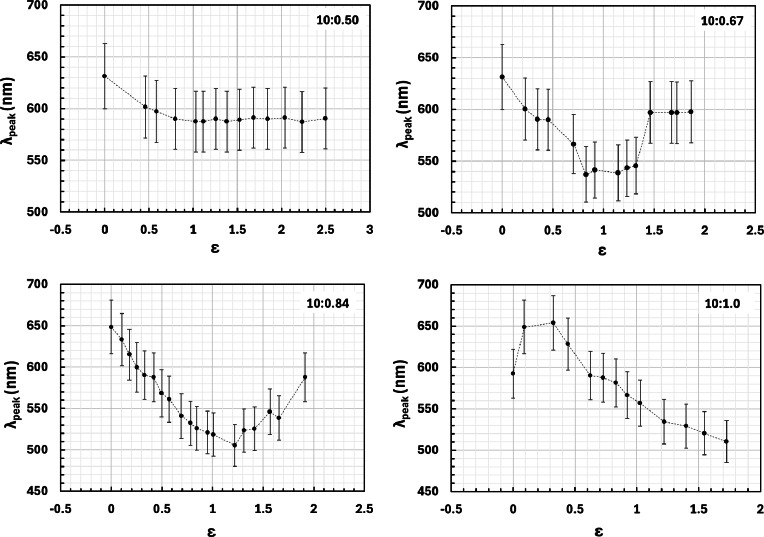
Peak reflected wavelength (λ_peak_) as a function of the extension of the PDMS/ss-ACLCE composite systems with the silicon elastomer base to curing agent ratios of 10:0.5, 10:0.67, 10:0.84, and 10:1.0.

Considering these, while interpreting the spectra and when possible, secondary peaks associated with the ACLCEs were analysed. With this, it was possible to plot the peak wavelength with the extension of the samples, for each of the PDMS silicon elastomer base to curing agent ratios (Fig. 5). Even so, at higher extensions, the effect of the background peak associated with the light source intensification, potentially caused by the reduction of light transmittance, associated with the PDMS matrix densification under imposed strain along the y-axis, rendered the ACLCE signal undetectable.

**Fig. 6:**
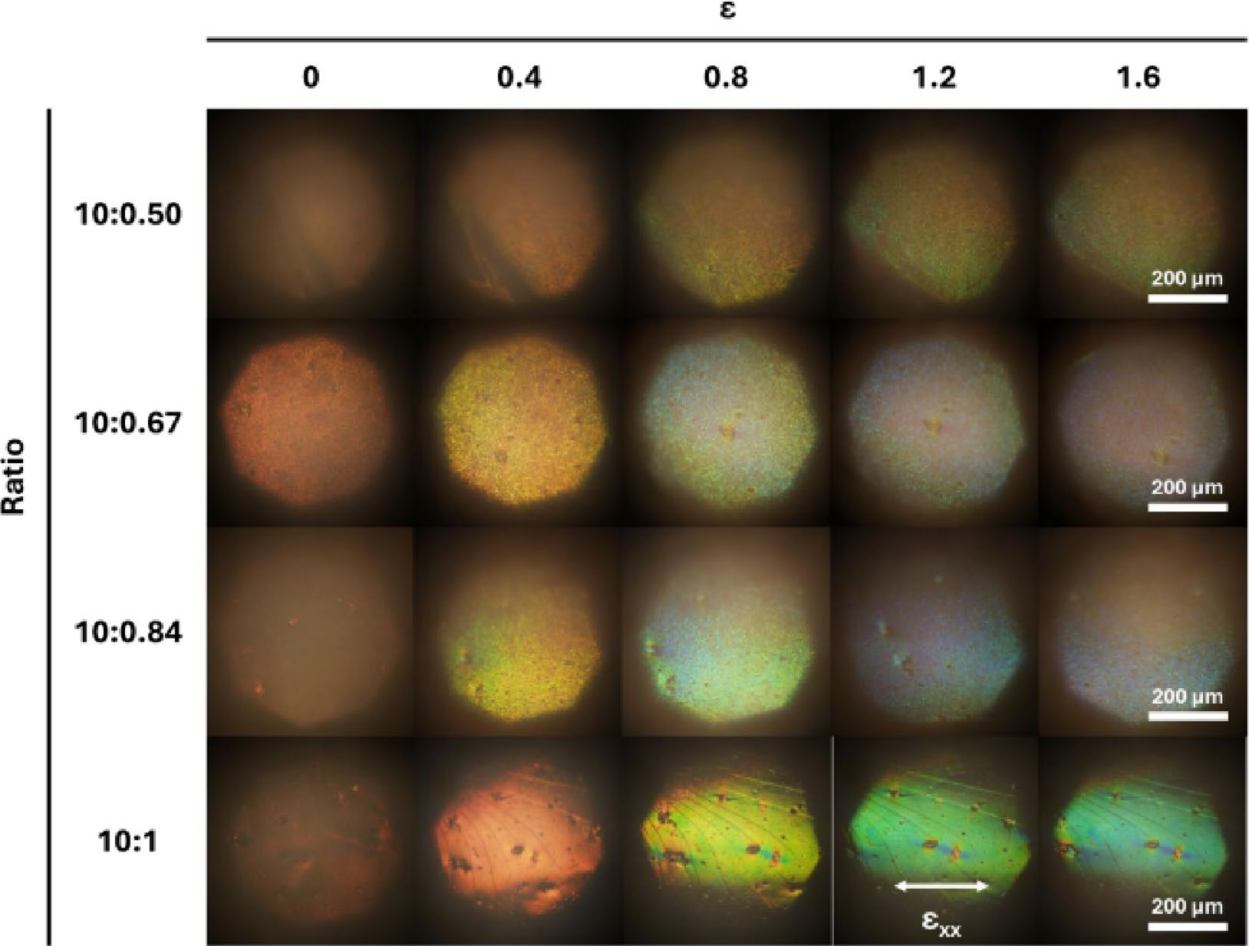
Optical microscopy images in reflection mode with parallel polarisers, demonstrating the mechanochromic response of PDMS/ss-ACLCE composite systems with the silicon elastomer base to curing agent ratios of 10:0.5, 10:0.67, 10:0.84, and 10:1.0 when submitted to tensile stress, at elongations of 0, 0.4, 0.8, 1.2, and 1.6. The direction of the tensile strain is represented by ε_xx_. The yellow hue present in most images is attributed to the light source.

The effect of the background light source was also observed on the obtained optical microscopy images, especially for the ratios of 10:0.50 and 10:0.67 (Fig. 6). The optical microscopy images obtained from the sample with the ratio of 10:0.50 presented a yellow hue for all extensions, which is in accordance with the spectra, which exhibited almost a linear behaviour with a wavelength around 590 nm after an extension of 0.75. In the sample with the ratio of 10:0.67, the yellow hue started to become more prominent at an extension of 1.2, corresponding to the sudden increase of the wavelength of the spectra to around 600 nm at around an extension of 1.4.

The PDMS/ss-ACLCE composite samples with the ratios of 10:0.84 and 10:1.0 produced near-blue reflection, reaching a wavelength of around 510 nm, and, as expected, considering that by applying tensile strain (ε_xx_) on the composite systems, a compression strain (ε_zz_) is consequentially applied to the ss-ACLCEs, for higher elongations, smaller wavelengths of the reflected light were observed, due to the reduction of the helix pitch. The relationship between the peak wavelength of the reflected light and the extension was in accordance with the results found in the literature^10^, especially for the samples with the ratios of 10:0.84 and 10:1.0.

Even so, in the optical microscopy images of the sample with the ratio of 10:0.84, the effect of the background light became prominent for an extension of 1.6, which is in accordance with the spectra, which exhibited a sharp increase in the wavelength after an extension of 1.2, reaching a wavelength of around 590 nm at an extension of 1.9. Therefore, the further potential of this formulation could not be assessed due to the effect of the background light source.

Only the sample with the ratio of 10:1.0 showed close to no impact of the background light source in the optical microscopy images. Furthermore, it showed a pronounced mechanochromic response with a consistent decrease in the wavelength with the increase of extension, supporting the potential of the ss-ACLCEs obtained through the free-fall dropwise addition method. It is important to point out that this sample fractured at an extension of 1.8, before achieving a full spectral shift by reaching 480 nm. Furthermore, the skewness observed in the graphic occurred due to an adjustment to a region where the mechanochromic response was more intense during the measurements.

## Conclusion

The goal of the present work was to develop mechanochromic sensors for application in strain detection, such as concrete crack propagation. For this effect, CLCE-based mechanochromic devices were obtained.

The free-fall dropwise addition method was considered to yield the best results, with no breakage or tailing of the spheres, coalescence, or disruption of the CLCs, which constitute effects that were observed for the remaining used methods for dropwise addition. Even if the obtained structures were semi-spheres, due to the gravitational settling, the observed mechanochromic response on the surface of the ss-ACLCEs was pronounced. Furthermore, the ss-ACLCEs proved to be compatible with PDMS embedding. The compatibility is highly dependent on the silicon elastomer base to curing agent ratio, which affects the rigidity of the PDMS films, and, therefore, the capability to transmit the deformation to the CLCs. Indeed, the best results were observed for the samples with the ratios of 10:0.84 and 10:1.0, and, even for those sample, it was not possible to perform a full assessment of their mechanochromic response, since the effect of the background light source became dominant for the samples with the ratio of 10:0.84 and since the samples with the ratio of 10:1.0 fractured before being possible to observe whether or not they would allow a full visible spectral shift. However, it is important to consider that the mechanochromic shifts were observed across a wide range of strains – until around 170% – therefore, future work should consist of employing more rigid transparent matrices to enhance the sensitivity of the devices to smaller strain variations.

## Supplementary Information

Below is the link to the electronic supplementary material.

Supplementary Material 1

## Data Availability

The datasets used and/or analysed during the current study are available from the corresponding author on reasonable request.
