# Supplementary material for: Mechanochromic cholesteric liquid crystal devices for mechanical strain detection
Source: Sci Rep. 2026 Jan 27;16:6298. doi: 10.1038/s41598-026-37723-4 (PMC12905114; doi:10.1038/s41598-026-37723-4)
Supplement: Supplementary file 1 — Supplementary Material 1 [file 41598_2026_37723_MOESM1_ESM.docx]

Mechanochromic Cholesteric Liquid Crystal Devices for Mechanical Strain Detection

Francisco Sousa ^a^, João Santos ^a^_,_ José F. Malta ^a^, João P. Canejo ^a^, Ana P. C. Almeida ^a,b *†^, Pedro L. Almeida ^a,c *†^

^a^ i3N/CENIMAT, Department of Materials Science, NOVA School of Science and Technology, NOVA University Lisbon, Campus de Caparica, Caparica 2829 - 516, Portugal.

^b^ LAQV/REQUIMTE, NOVA School of Science and Technology, Caparica 2829-516, Portugal.

^c^ UnIRE/ISEL, Mechanical Engineering Department, ISEL, Polytechnic University of Lisbon, Rua Conselheiro Emídio Navarro, 1, 1959-007 Lisbon, Portugal.

^†^ These authors contributed equally to this work.

^*^ Corresponding authors: [pedro.almeida@isel.pt](mailto:pedro.almeida@isel.pt)

[ana.almeida@fct.unl.pt](mailto:Ana.almeida@fct.unl.pt)

**SUPPLEMENTARY MATERIAL**

**Dropwise addition method:** To maintain the magnetic stirring bar stable, a rotation speed of at least 100 rpm was required. However, at such speeds, due to insufficient structural integrity, the ACLCE droplets ended up flattening or collapsing entirely, and even by adjusting the crosslinking reaction timing with the beginning of the dropwise addition process, either the droplets started splitting, and coalescing, due to insufficient crosslinking and non-uniform stirring speed distribution (Figure S1a), or started tailing, due to excessive crosslinking. Even so, the edges of the beads that were not in contact with the coalesced mass exhibited red structural colour with a blue shift when submitted to compression (Figure 3b), which proves the potential of the ACLCE beads for mechanochromic sensors. The distribution of structural colours observed in Figure S1b occurs due to a non-uniform compression. The edge in contact with the coalesced mass did not exhibit any structural colour, probably due to the disruption of the molecular order. In any case, considering the coalescence, the magnetic stirring method was discarded.


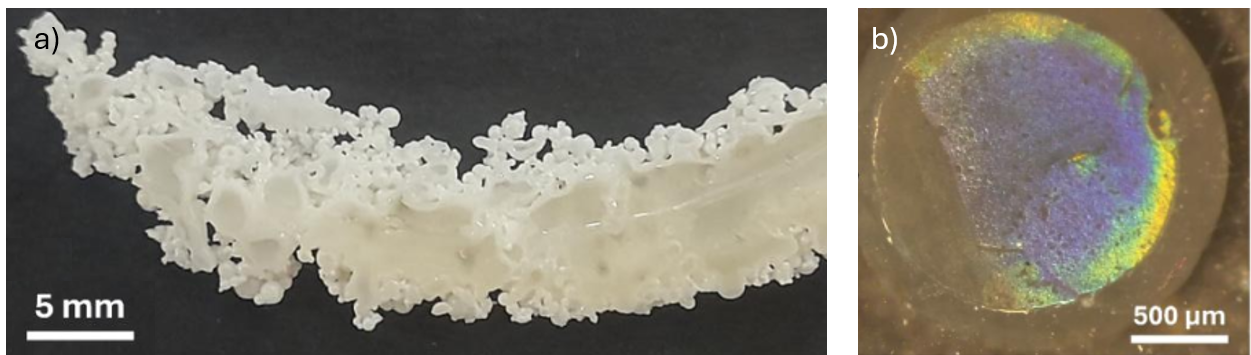


Figure S1 - a) Coalesced mass of ACLCE beads obtained through dropwise addition with magnetic stirring, and b) coalesced bead structural colour after compression.

Mechanical stirring was then employed to allow a slower rotation speed, uniform stirring speed distribution, and control of the flow type. As intended, with mechanical stirring with a pitched-blade impeller at a speed of 40 rpm, and by dispensing in a time window between 30 and 50 min post-synthesis, droplet shape disruption upon dropping did not occur. However, due to the low viscosity, the stirring motion and the generated axial flow were still enough to deform the shape of the droplet in the silicon oil and led to coalescence. Nonetheless, the obtained beads exhibited a slight mechanochromic response, with a shift from transparent to a faint light blue structural colour (Figure S2). A potential explanation for this weak response is the utilised time window, which led to the crosslinking reaction already being underway at the time of dropping into the oil bath, resulting in insufficient reaction time in the silicon oil for a full molecular ordering.


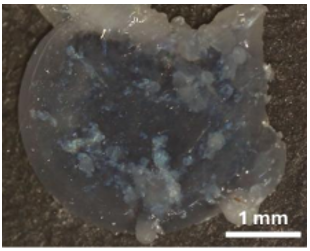


Figure S2 - Coalesced ACLCE bead obtained through dropwise addition with mechanical stirring, using a pitched-blade impeller. The bead exhibited a faint light blue structural colour after compression.

In an attempt to overcome this limitation, and considering that the utilized time window was already optimized to avoid the disruption of the bead shape, an alternative formulation was tested to increase the workable time window – dilution of the catalyst from [1:10] to [2:10] (V/V) toluene-to-catalyst ratio to achieve a final concentration 0.085, instead of 0.17 mL/mL.

This method successfully delayed the gelation. However, slower crosslinking led to an increase in coalescence. For the case of the diluted catalysing solution, even though the material showed some small points with red structural colour, the structure showed poor mechanical integrity. As a result, it could not be employed for mechanochromic applications. For this reason, the dropwise addition method with a pitched-blade impeller was excluded from further analysis.

To overcome the coalescence, an anchor-type impeller was used to generate a tangential flow, which, combined with the used rotation speed ramp of 10 to 70 rpm, allowed the droplet to be immediately dropped after synthesis with no collapse of the shape. As the crosslinking reaction progressed and the solvent evaporated, the beads started to deposit at the bottom. The stirring was maintained for 70 min until the gelation was complete, and after 2 hours, the obtained beads exhibited red structural colour. However, after 1 day, the structural colour had completely disappeared, and the beads showed no mechanochromic response. A possible reason is that the applied speed ramp caused a slower solvent evaporation. When the red structural colour was observed, the solvent had not yet fully evaporated, and, as evaporation continued, the molecular ordering of the ACLCEs changed, leading to the disappearance of the visible structural colour.

Agitation was included in the methods mentioned above to avoid the flattening of the base of the beads caused by the gravitational settling once dropped. However, as seen before, even coalesced beads exhibited structural colour and mechanochromic behaviour. Furthermore, it was observed that the agitation could lead to the disruption of the molecular order. Considering this, the free-fall dropwise addition method was employed.

As imagined, the obtained beads presented a semi-spherical shape, obtained due to the flattening of the base, combined with the shaping of the surface, caused by interfacial tension. The beads had an average diameter of 1.08 mm and presented vibrant red structural colour and a pronounced mechanochromic behaviour on their upper surface with a shift to a blue structural colour (Figure S3a). The directionality of solvent evaporation can explain this phenomenon. Unlike the dropwise addition with agitation, in which the constant stirring ensures that the solvent evaporates evenly throughout the entire surface area of the beads, in this case, toluene must have evaporated preferentially through the upper exposed surface of the beads. Since the molecular organisation and cholesteric self-assembly are dependent on the deswelling through solvent evaporation, the surface-localised solvent evaporation led to the formation of structural colour on the surface of the beads and potentially led to an enhanced ordering when compared to the remaining structures obtained in this work (Figure S3b). The pronounced mechanochromic response of the semi-spherical ACLCE (ss-ACLCE) beads obtained through the free-fall dropwise addition method proves the potential of this structure for sensory applications, where a distinct shift in colour, in response to a slight mechanical stimulus, is required.


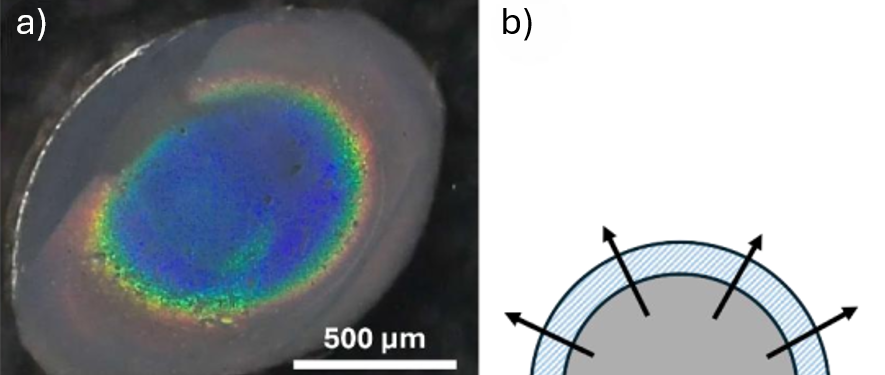


Figure S3 - a) Structural colour of an ACLCE bead obtained through free-fall dropwise addition, upon compression; b) Schematic of the surface-localised solvent evaporation.

**Moulding method:** Parallel to the dropwise addition method, the ACLCE precursor solution was directly poured into a cylindrical PLA mould (Figure S4). To seal off the bottom of the mould, a bar of PTFE was placed on the bottom, and parafilm was placed between the mould and the bar. The 3D ACLCE cylindrical structures were stored for 1 day at room temperature, after which they were demoulded. The cylindrical shape was chosen to achieve a more uniform stress distribution when compressing the structure.


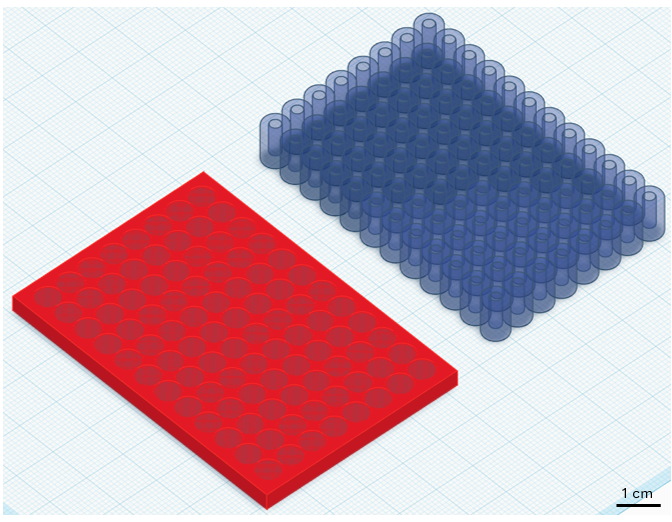


Figure S4 - 3D design of the cylindrical PLA mould for ACLCE shaping. The mould was designed with a hole in the cylindrical section to inject the solution and facilitate demoulding.

From the moulding method, 3D ACLCE cylindrical structures with a 6.83 mm diameter and a 1.9 mm thickness were obtained. When compressed, the samples displayed a weak mechanochromic response (Figure S5).


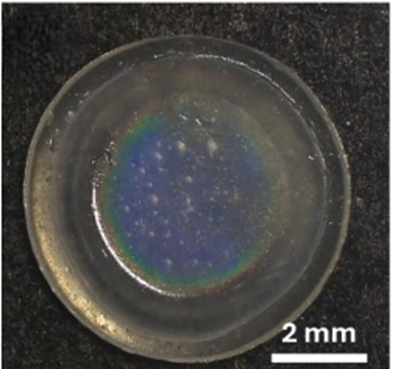


Figure S5 - Structural colour of an ACLCE cylindrical structure obtained through moulding, upon compression.

Considering that in a comparable setup, it was observed that an increase in thickness led to a decrease in the quality of molecular alignment [10], it can be assumed that the higher thickness of the cylindrical structures led to the weak mechanochromic response. Considering these results, the cylindrical ACLCE structures were not further tested.

Following their production, the ss-ACLCE beads were washed with water and soap to remove the silicon oil. However, after the washing step, the mechanochromic response disappeared. This possibly happened due to the swelling of the beads, leading to an alteration of the molecular ordering. To overcome this issue, the ss-ACLCE beads were reswelled with toluene and dried, which ended up recuperating the original properties of the beads by enabling molecular mobility and ordering. It is relevant to mention that the same process was attempted on the remaining types of beads to assess whether it could improve the mechanochromic response. However, no improvement was achieved, demonstrating that the reswelling with toluene only works on samples that already exhibited mechanochromism.

**Photo-initiator and UV curing:** In the materials and methods section of this work, it was mentioned that the photo-initiator Irgacure 651 was added to the formulation of ACLCEs. This was done to adjust the rigidity of the obtained structures, if necessary. However, considering that samples with the required mechanical properties were obtained, no UV curing was performed.
